# Climate change impact on ecosystem functions provided by birds in southeastern Amazonia

**DOI:** 10.1371/journal.pone.0215229

**Published:** 2019-04-11

**Authors:** Leonardo S. Miranda, Vera L. Imperatriz-Fonseca, Tereza C. Giannini

**Affiliations:** 1 Instituto Tecnológico Vale, Belém, Pará, Brazil; 2 Universidade Federal do Pará, Belém, Pará, Brazil; CNRS - Universite de Pau et des Pays de l'Adour - E2S UPPA, FRANCE

## Abstract

Although the impacts of climate change on biodiversity are increasing worldwide, few studies have attempted to forecast these impacts on Amazon Tropical Forest. In this study, we estimated the impact of climate change on Amazonian avian assemblages considering range shifts, species loss, vulnerability of ecosystem functioning, future effectiveness of current protected areas and potential climatically stable areas for conservation actions. Species distribution modelling based on two algorithms and three different scenarios of climate change was used to forecast 501 avian species, organized on main ecosystem functions (frugivores, insectivores and nectarivores) for years 2050 and 2070. Considering the entire study area, we estimated that between 4 and 19% of the species will find no suitable habitat. Inside the currently established protected areas, species loss could be over 70%. Our results suggest that frugivores are the most sensitive guild, which could bring consequences on seed dispersal functions and on natural regeneration. Moreover, we identified the western and northern parts of the study area as climatically stable. Climate change will potentially affect avian assemblages in southeastern Amazonia with detrimental consequences to their ecosystem functions. Information provided here is essential to conservation practitioners and decision makers to help on planning their actions.

## Introduction

The average global surface temperature has increased by approximately 0.8°C during the last century and is expected to continue increasing [1]. Species have always reacted to climatic changes throughout their evolutionary history [2–4]; however, currently, the main concern is the unprecedented rapidity of the observed changes [1]. Although, until now, habitat loss and fragmentation have represented the highest threat to biodiversity [5,6], some studies have suggested that climate change is likely to outweigh habitat loss as a global threat in the coming decades [7]. In fact, even though climate change constitutes its own set of risks, it may interact with habitat loss and increase shifts in species distributions, extinctions, and hence compositional changes in communities [8]. This potential distribution reshuffling of biodiversity may affect the structure, dynamics and functioning of ecosystems and the contributions they provide [9].

Nature’s contributions to people (NCP) is the concept of “the benefits that people derive from nature [to provide] a good quality of life” proposed by the Intergovernmental Platform on Biodiversity and Ecosystem Services [10,11]. Similar to the more popular ecosystem services concept [5], nature components (biodiversity) interact in complex processes that control flows of energy, nutrients and organic matter (ecosystem functioning) that, in turn, produce environmental goods and services (e.g., clean air, fresh water, climate stabilization) that contribute to health and human well-being.

Birds are good biological indicators of climate change impacts on NCP, since they occupy all terrestrial habitats, consume virtually all type of resources [12] and therefore provide key ecosystem functions and services such as pollination, seed and nutrient dispersion, predation, and scavenging [13]. Besides, they are one of the most well-known and studied groups, with a huge amount of data available. Nonetheless, even for birds, there is a deficiency of data in terms of describing and identifying the biodiversity of the Amazon rainforest and providing high-quality species distribution data [14,15]. For example, according to the last available survey based simply on georeferenced occurrence maps [16], this biome harbours 1778 resident bird species. However, this number continues to rise as new species are discovered [17], and to date, large areas have not been inventoried in Amazonia [14].

Amazonia is a complex biome, and, just in floristic terms, it consists of areas covered either by forest (FT; *terra-firme*), periodically inundated forested environments (*várzea*, *igapó*), as well as open-area vegetation patches (OAV; *cerrado*, *campinas*/*campinaranas*, *cangas*). This environmental heterogeneity is one of the leading factors responsible for its high species richness [18,19]. Moreover, despite being recognized as providing key NCP [20], Brazilian Amazonia has already lost approximately 19.6% of its forest [21,22]. Land-use change caused by agricultural expansion, logging, mining and energy production are responsible for more than 90% of Brazil’s total greenhouse gas emissions [23]. Therefore, the rapidity of environmental changes has increased the urgency of collecting, organizing and analysing biodiversity data to guide decision-making in conservation. Moreover, the NCPs are essential to human activities, and a better understanding of the range of ecosystem responses to climate change will help to improve the determination and the implementation of effective ecosystems management in a manner that promotes resilience [24]. Although several studies have found a strong negative impact of forest fragmentation on the biodiversity in Amazonia [6,8,25], very few studies have evaluated how species will be affected by climate change in the near future [26,27] and fewer studies have examined the consequences of climate change on functional diversity [26].

In this study, we gathered extensive species occurrence data representative of southeastern (SE) Amazonia to assess the potential climate change impact on avian assemblages. Our work encompasses 501 species (representing more than 50% of the known avian diversity of the focal area [17] and 199,250 occurrence records (S1 Table). Using Species Distribution Modeling (SDM), we analysed how different scenarios of climate change could affect the pattern of species distributions and assemblage compositions. By grouping species based on their main diet (frugivores, insectivores, nectarivores and others [28]) as a proxy to NCPs (seed dispersion, pest control and pollination), we were able to indicate the most susceptible functional group to climate change. In addition, we evaluated the future effectiveness of current Protected Areas (PAs; Conservation Units [CUs] and Indigenous Lands [ILs]) and presented focal areas for different conservation planning considering the dynamics of climate scenarios.

## Material and methods

### Study system

The species list was derived from those recorded in Carajás, currently one of the biologically best known areas in the SE Amazonia due to over three decades of periodic inventories and sporadic observations [29,30]. It was updated from Vale’s internal database (*bdbio*), internet repositories (e.g., http://wikiaves.com.br and http://xeno-canto.org) and Museu Paraense Emilio Goeldi ornithological collection, achieving 620 species. The projections and analysis focuses mainly on SE Amazonia, an area between the right margin of Tapajós and Juruena Rivers (59°W) and the Amazonia east limit (44°W), and from Marajó island (0°) to the Amazonia south limit (14°S). The south and east limits of this area correspond to the Amazonia-Cerrado transition zone, encompassing FT and OAV environments, and it is naturally close to the climatic limit of tropical forests [31,32]. In fact, several climate model simulations confirm that SE Amazonia is the most sensitive region to global climate change [33,34] and it has the most deforested area and the highest deforestation rates considering all Amazonia [22]. It is important to mention that our set of species corresponds to *ca*. 63% of the total known avian taxa of the focal area, and, although some avifauna turnover may occur between the interfluves of major rivers, most species have wide distributions and less than 1% represent taxa exclusively endemic to the respective interfluves. In addition, the list includes FT and OAV species, which allow testing if and how these groups will potentially respond differently to climate changes. Moreover, to assess changes in ecosystem functioning, we also assign each species to one of three trophic guilds based on the main diet of the species [28]: frugivores (FR [seed dispersion]), insectivores (IN [pest control]) and nectarivores (NE [pollination]). Species with other diets (e.g., omnivores, granivores, carnivores, and scavengers) were assigned as "others" (OT).

### Data collection

As above mentioned almost all species analysed present wide distributions, thus the total known distribution area of each species in the Neotropics was used and then later projected to the focal area. Our occurrence data were compiled from the open access platform Global Biodiversity Information Facility (available from http://gbif.org; accessed 19 July 2017), using the R package rgbif [35]. We applied a quality control to minimize sample bias by using only georeferenced data, ensuring a minimum distance of 10 km between consecutive presence records (lower spatial aggregation). We also excluded dubious/disparate records, i.e., those that were clearly outside the expected bird distribution, grounding our decision in the Handbook of the Birds of the World and BirdLife International [17]). Our database includes 228,298 nonduplicate presence records (ranging from 9-1297/taxa) for 547 species (introduced, migratory and aquatic species were excluded). Of these species, 23 are Brazilian endemic, 49 are facing some degree of threat [36–38] and only four species have a limited availability of occurrence data (<15 records; all endemic and/or rare). Species taxonomy follows the latest updated checklist of the Brazilian Ornithological Records Committee [39].

Current climate data and projections for 2050 and 2070 were obtained from the WorldClim database (available at: http://worldclim.org; accessed 24 November 2016) at a resolution of 5 arcmin (~10km at the equator) for the Neotropical region (spatial limits of 110° to 30°W; 25°N to 60°S). This resolution was chosen due to the high computational power demanded for modelling such a high number of species, their broad distributional area and high number of scenarios. After performing a pairwise Pearson correlation test for all 20 variables (19 bioclimatic and altitude) to remove those that were highly correlated (r>0.85), we selected 11 as our predictors: annual mean temperature, mean diurnal range, temperature seasonality, temperature annual range, mean temperature of warmest quarter, annual precipitation, precipitation of driest month, precipitation seasonality, precipitation of warmest quarter, precipitation of coldest quarter and altitude. Future climatic projections were derived from general circulation model (GCM) simulations from the Community Climate System Model (CCSM4), a well-known and frequently used GCM (e.g., [40,41]). In addition, among the various GCMs, the Amazonia rainfall and its seasonality have highly variable bias (which account to the uncertainty in forecasting future atmospheric CO2 concentration and climate change), yet the CCSM4 is one of the models that best represent the main climatic drivers on the region [42,43]. Three representative concentration pathway scenarios (RCPs 2.6, 6.0 and 8.5) were projected, representing a broad range of climate outcomes from the most to the least optimistic scenario.

### Species distribution modelling

The SDMs were estimated by running two different algorithms within the biomod2 package on R [44]: generalised linear models (GLM [45]) and maximum entropy (Maxent [46]). We generated three sets of pseudo-absence data containing ten times the number of presence data points randomly distributed for each species [47]. The data sets were then partitioned so that 80% were used to calibrate the models, while the remaining 20% were used for the evaluation. Model performance was assessed with the True Skill Statistics (TSS; threshold-dependent [48]) and the receiver operating characteristic curve analysis (ROC; threshold-independent [46]), this procedure was repeated ten times for cross-validation, and the models with poor performance (TSS<0.5) were eliminated from the ensemble process. We also report sensitivity (percentage of true positives), specificity (percentage of true negatives) and assessed the predictor variable importance using the tables of permutation importance.

Finally, we summarized the ensemble of predicted species distributions (two algorithms x three pseudo-absences x models with TSS>0.5) using the committee average method, where probabilities from different models were first transformed into binary according to a threshold (here, the TSS cut-off threshold) and then averaged [44]. In the end, seven consensual maps were generated per species (one for each scenario: current, and RCPs 2.6, 6.0, 8.5 for 2050 and 2070).

### Analyses

First, our continuous probability consensual maps were converted into a binary classification by selecting threshold values that maximized the TSS (cutoff; S1 Table). The projected occurrence area was calculated by multiplying the cell area (10x10 = 100km^2^) with the cell count. Species with projected current occurrence areas <100 grid cells were excluded from the analyses, to avoid overestimating the predicted effects within the focal area. Therefore, the final dataset included 501 species and 199,250 records (S1 Table). Our data include species that occur in both FT and OAV environments, and we treat them separately. The total species richness (SR) was computed by stacking the maps of all individual species for each scenario (and for each algorithm to estimate uncertainties, see next). Uncertainties were calculated as the contribution of different sources (here, the algorithms and future scenarios) to variation around the consensus maps, through the proportional sum of squares of each cell in relation to the total sum of squares using the SR maps as response [49]. We estimated the species range change dynamics (in terms of area gain/loss) based on the differences between the projected area for each future climate scenario and current. To highlight the impact on ecosystem functioning, these results were grouped by the trophic guild (see above) to reflect the most affected function in the study area. We also assessed the future effectiveness of current PAs by accounting for the differences in the estimated SR in each PA over the different climate scenarios. Although there are 57 PAs (37 CUs and 20 ILs) in the focal area, we grouped them in 13, since they form continuous blocks of protected area and were considered together. Thus, for this analysis we kept only blocks with an area greater than 1,000 km^2^ (S2 Table; data for Brazilian PAs available from Instituto Chico Mendes de Conservação da Biodiversidade: http://icmbio.gov.br). Finally, for the FT species only, we calculated the size of climatically stable areas considering grid cells containing at least 20% of each trophic guild and with an increment of +10% (until 80%) in each scenario. We first stacked each series of percentage/guild on one binary map, and stacked this map with each scenario to show the suitable habitats across scenarios. Areas were considered climatically stable if the guilds were predicted to be present in six to seven out the seven scenarios. Also, a deforestation layer (data for Amazon for the year 2015 available from Instituto Nacional de Pesquisas Espaciais [22]: http://www.obt.inpe.br) was downscaled from the original resolution (60 m) to the topoclimatic variables resolution (10 km) and then, overlaid to create a final decision-making oriented map. The climatically stable areas, outside PAs, that potentially will maximize suitable habitats for biodiversity and having natural vegetation were considered important to conservation, and the same areas with no natural vegetation, important for restoration programs. All analyses were performed using R [50] and QGIS [51].

## Results

The quality of the models, according to TSS (0.68 ± 0.11) and ROC (0.90 ± 0.05; all values above 0.80), had a high levels of accuracy (S1 Table), indicating good model fit. Temperature Seasonality (BIO4, for 80% of the species), Annual Mean Temperature (BIO1, for 67%) and Mean Temperature of Warmest Quarter (BIO10, for 46%) were important drivers (mean importance > 30%) for many species distributions; and all species (except for *Myiozetetes cayanensis*) have at least one predictor variable with importance greater than 30% (S1 Table). The sum of squares obtained to each cell showed distinct contributions to variation around the SR maps (S1 Fig). Algorithms are responsible for the greatest proportion of variation (FT median [min-max] = 79% [5–100%]; and OAV median [min-max] = 87%, [1–100%]), widely spread in the study area. The proportion of variation attributable to future scenarios was lower than the former (FT median [min-max] = 62% [1–100%]; and OAV median [min-max] = 41%, [1–100%]).

### Species richness and range change dynamics

**Fig 1:**
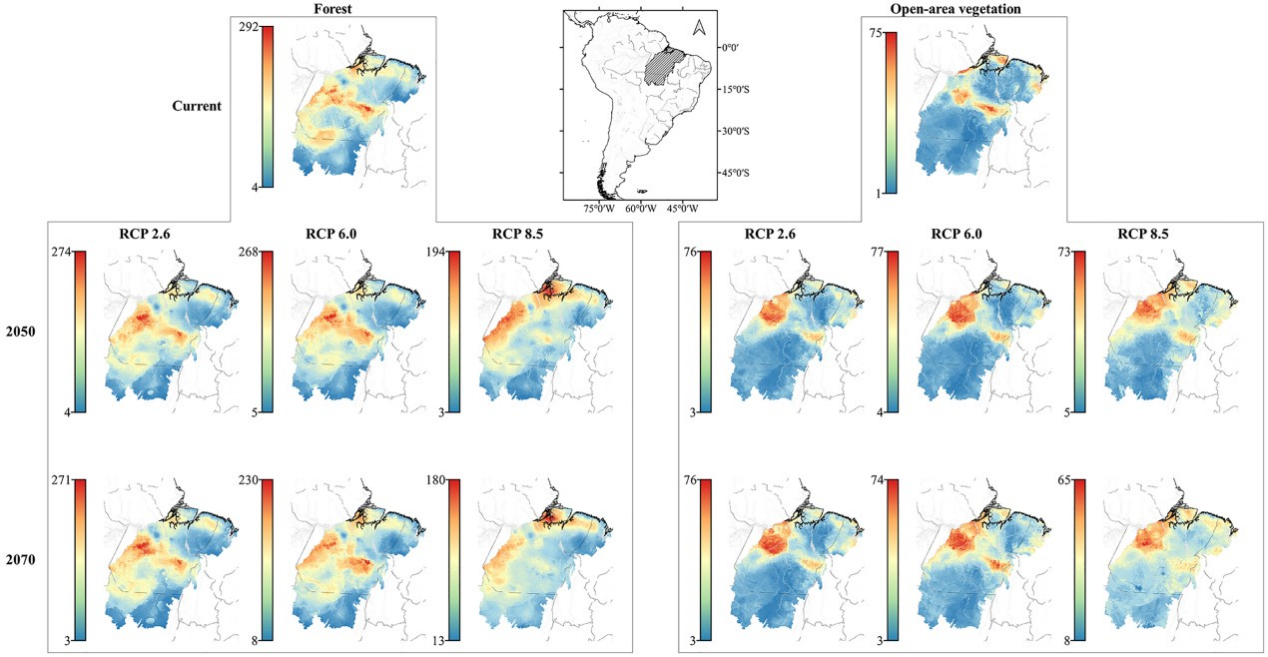
Maps of projected species richness. Avian assemblage for current and future (2050 and 2070) climate change scenarios (RCP 2.6, 6.0 and 8.5). Hotter colours represent higher numbers of species with forecasted suitable habitats for each scenario.

For FT species (N = 382), our current projections indicated high SR in the centre and western portion of the focal area (≥50%, reaching 76% by cell), and low SR in the south eastern portion (<30%). Under the future scenarios, climate change may result in significant loss in SR (reaching 47% at its maximum in the worst case scenario) besides a distributional shift from west-central richest area to north towards the Marajó island (Fig 1). The current projected areas with high SR for OAV species (N = 119) were the central portion of focal area (reaching 59%), and east of Marajó island (46%). Our future scenarios forecasted a northward shift from the core occurrence region (not shown; see the Discussion) resulting in increased SR (Fig 1).

**Fig 2:**
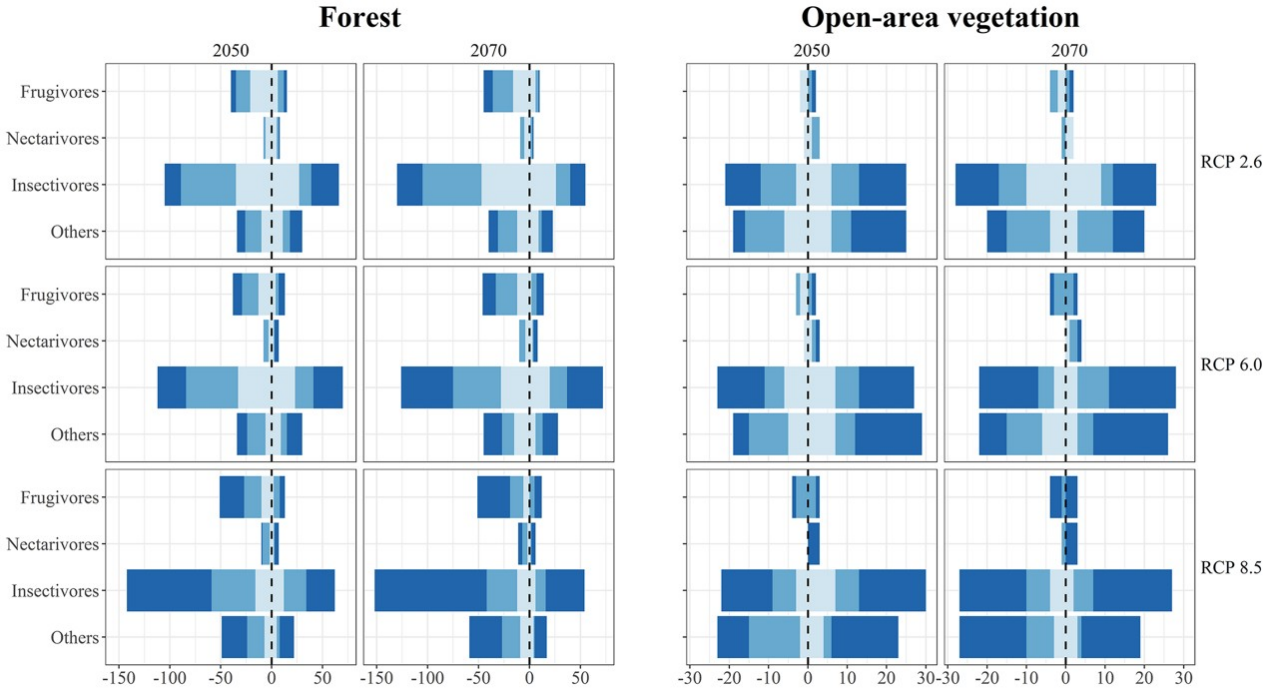
Potential climate change effects on forecasted occurrence area per species. Negative values represent the number of species predicted to lose suitable habitats under different scenarios at each guild class. Positive values represent the number of species predicted to gain suitable habitat. Light blue: ≥20%, blue: ≥ 50%, and dark blue: ≥ 90%.

**Fig 3:**
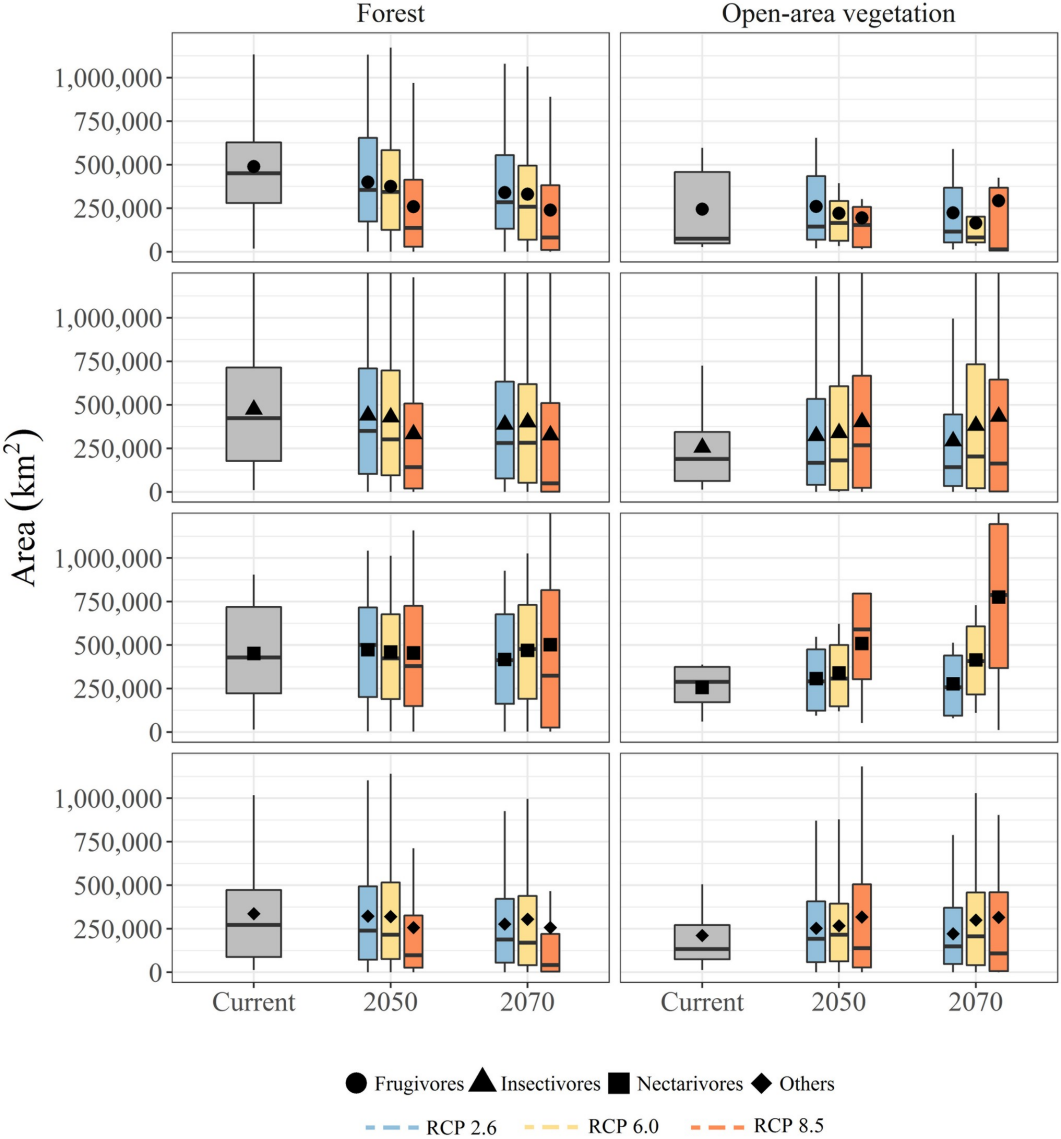
Potential change in mean area per trophic guild. Mean area for frugivores (circles), insectivores (triangles), nectarivores (squares), and other guilds (diamonds). Centre lines show the medians; box limits the 25%-75% percentiles in current (gray) and different future (2050 and 2070) climate change scenarios—RCP 2.6 (light blue), RCP 6.0 (orange), and RCP 8.5 (red); and whiskers 1.5x interquartile range of the data.

Our results indicate that a greater number of species were projected to show a range loss rather than a range gain (Fig 2). Independent of trophic guild, approximately 187 (49% for FT) and 43 (36% for OAV) species will potentially lose ≥20% of their current suitable area in the next 20 years in the most optimistic scenario (2050; RCP 2.6), and between 72 (19% for FT) and 13 (11% for OAV) species will no longer find suitable habitat in the focal area in the worst case scenario (RCP 8.5; 2070; Fig 2, S3 Table). In absolute numbers, FTIN accounts for most of the projected loss of geographic range size (out of the 219 species between 105 [48%] and 152 [69%] are expected to lose at least 20% of current projected suitable habitats depending on the scenario), but proportionally the FTFR species will potentially be more negatively affected (out of the 66 species between 38 [58%] and 51 [77%] are expected to lose at least 20% of current projected suitable habitats depending on the scenario). Most of the species projected to gain geographic range sizes are from OAV group with an average increase of 234% (SD 467%; Fig 3, S3 Table). All guilds, for FT species, will potentially experience a gradual decline on mean range size (Fig 3), except for FTNE from which four species presented an increase of range size far greater than 100% pulling up the mean (S3 Table). Our models also projected that OAVFR species would experience a decline on mean range size (from near 245,000 km^2^ in current projections on average, to less than 195,000 km^2^ considering the RCP 8.5 for 2050; Fig 3, S3 Table), while for the remaining groups, an increase (Fig 3, S3 Table).

### Effectiveness of current protected areas and priority areas

**Fig 4:**
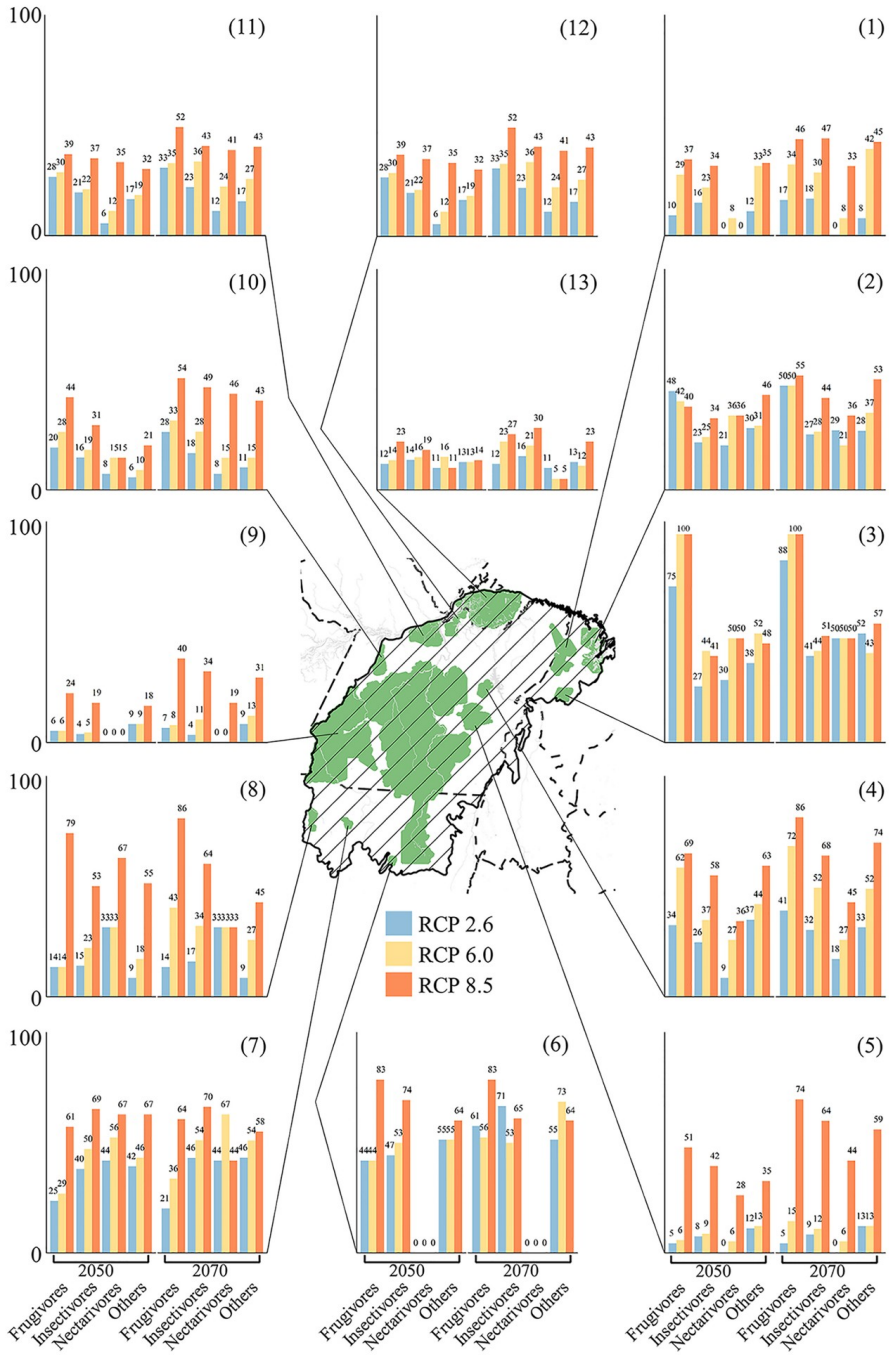
Percentage of species predicted to disappear in each protected area (PA). Each bar plot represents the percentage of species loss in relation to current projected species richness and assuming no species turnover. The central map and numbers therein indicate the PA names: (1) Gurupi Mosaic; (2) APA Baixada Maranhense; (3) IL Guajajara; (4) IL Parakana; (5) Carajás Mosaic; (6) ESEC Rio Ronuro; (7) IL Kayabi; (8) IL Erikbakts; (9) Terra do Meio Mosaic; (10) FLONA Tapajós; (11) RESEX Renascer and Verde para sempre; (12) RESEX Gurupá-Melgaço and FLONA Caxiuanã; (13) Marajó Mosaic. For more details see S2 and S4 Tables.

The 13 mosaics of PAs in the focal area potentially protect on average 53% (SD 29%) of the species, according to current projections (S4 Table; the lowest values occurred at the most southern PAs [ESEC Rio Ronuro, 13% and IL Rikbaktsá, 15%]; and the highest values occurred at the centre [Carajás Mosaic, 82%] and western [Terra do Meio Mosaic, 98%] PAs). Under future scenarios, approximately 34% (SD 14%) of the current forecasted species in each PA will potentially not find suitable habitats (disregarding species turnover), and the projected species loss displayed distinct patterns depending on the trophic guild, scenario, and PA position and size (Fig 4, S4 Table).

**Fig 5:**
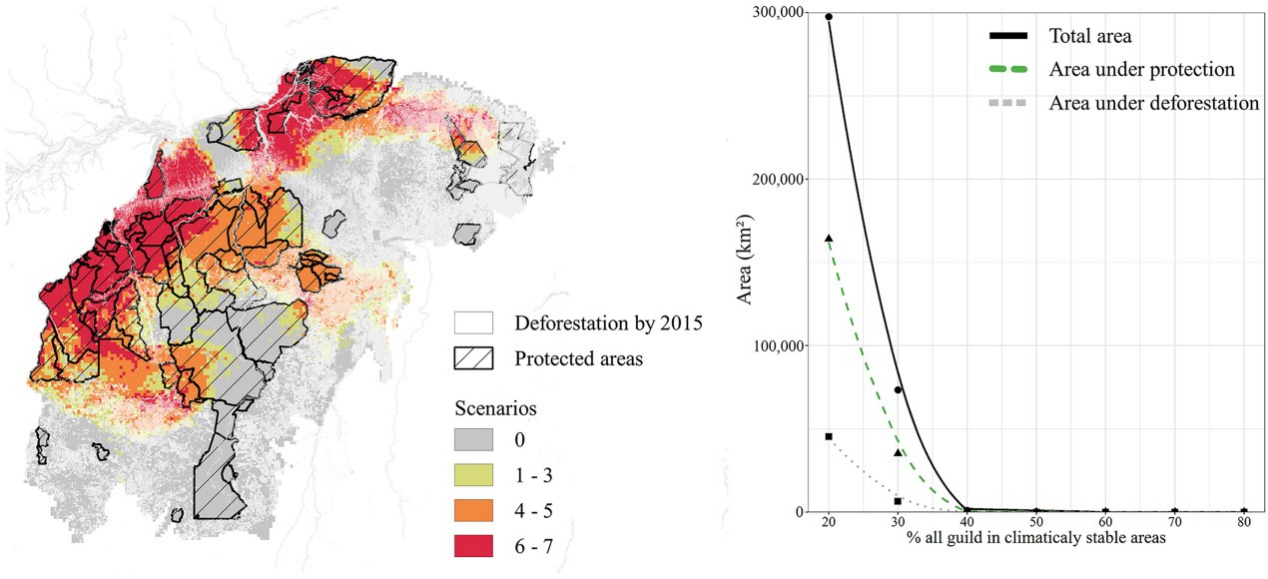
Potential climatically stable areas for conservation actions. Map indicating climatically stable suitable areas for at least 20% of each trophic guild across seven climatic scenarios. Colour scales represent the overlap of the models. All the priority areas are plotted with currently existing Protected Areas (PAs) and deforestation layers. Graphs represents the amount of climatically stable suitable areas for different percentages of a minimum guild class (solid line); the amount of area overlapped with PAs (dashed green line) and overlapped with deforested area (dotted grey line).

Our analyses suggest that the majority of the northern and western parts of the focal area are climatically stable suitable habitats for FT species, indicating that suitable habitats coincided with six to seven of the seven scenarios (Fig 5). Climatically stable grid cells which were considered suitable habitats for at least 20% of each trophic guild correspond to 297,500 km^2^. Nearly half of this area is within PAs (164,000 km^2^ [55%]), yet another significant part has native vegetation but unprotected (88,100 km^2^ [30%]) or overlaps with degraded areas (45,400 km^2^ [15%]; Fig 5). Also, there is a drastic reduction in the stable area when considering a higher proportion of each trophic guild per grid cell, for instance, climatically stable cells that were considered suitable habitats for at least 30% of each trophic guild correspond to only 73,400 km^2^ (a decrease of about 75%) and there is no single cell that maintains at least 50% of each guild in most scenarios (Fig 5).

## Discussion

Climate change will impact the biodiversity across the globe [7] and it is important to forecast the effects not only on range shifts but also on the functionalities [52]. Through our SDMs we show that avian assemblage from SE Amazonia will be potentially strongly affected by climate change in the near future, even under the most optimistic scenario. As expected, species occupying different habitats will respond differently, but invariably frugivores will be more negatively affected. Considering only FT species, currently, the centre and western portion of SE Amazonia are potentially the richest areas, and future projections suggest a northern shift with an expressive depletion in SR. The OAV species have a disjunct distribution, where the occurrence core is in the “dry diagonal” (out of the scope of this work) and isolated patches of the Amazonia [53]. As our study system includes a transition zone encompassing both environments (FT and OAV), the OAV species occur naturally at low numbers (<30% in almost all areas) but potentially could reach 46% east of Marajó Island and 59% in patches at the central and northwestern portion of the focal area based on our current projections. Under the future scenarios, potential SR was predicted to increase due to a northward shift (from the core region, not shown) that becomes uniform along all of the focal area, maintaining its maximum value (~55%) at the northwestern portion.

According to our results, the majority of species were projected to show a range loss. In addition, in terms of trends in mean area change per trophic guild, the FT species presented a decrease in the mean projected area under all future scenarios, resulting in a progressive range retraction through time. It is worth pointing out the range expansion in some FT species that exceed in more the 100%. This could be due to the fact that SE Amazonia represents the distribution margin of some FT species and may not express its climatic affinities precisely [54]. However, it is also likely that other factors besides climate, such as those related to species ecological characteristics, could play a role in the distribution ranges. Therefore, finer resolution models and/or the inclusion of species-specific environmental factors may be needed to address these issues. Conversely, the OAV species presented an overall trend to increase progressively their distributional range. We hypothesized that FT species would have the greatest impact, and likely, climate change would favour OAV species, based on the expected "savannization" process [32,33,55] (but see [56]). Our models partially corroborate this idea, showing an increase in mean area of OAV species, except for OAVFR species. Based on our models, frugivores seem to be the most sensitive functional group (FTFR mean area loss ranging between 18.2% [2050; RCP 2.6] and 51% [2070; RCP 8.5], and OAVFR ranging between 9.7% [2050; RCP 6.0] and 32.8% [2070; RCP 6.0]) for the study area as a whole.

In terms of current effectiveness of PAs based on SDM, our results are in accordance with those from recently analysed Brazilian protected areas [57,58]. Here, we also provide an evaluation of future effectiveness of the currently established PAs for avian assemblages in SE Amazonia. Our SDMs project a significant loss of species considering future warming climate scenarios in the focal area, and this is even worse considering each PA apart, in which species loss can reach 100% (e.g., Frugivores in IL Guajajara, RCP 6.0 and 8.5). In fact, considering future effectiveness based only on the differentiation between current and future mean temperatures, it was estimated that approximately 19–67% of all current protected areas in Brazil will potentially not have similar temperature regimes in the future [40]. In addition, the frugivores are the trophic guild class that shows the strongest loss (ranging between 5–100% species loss, in Carajás Mosaic, RCP 2.6, 2050, and IL Guajajara, RCP 6.0, 2050, respectively), and other studies have already suggested this guild is more extinction-prone [59]. Frugivores play a key role in providing seed dispersal services [60], mainly in tropical rainforests (70–94% of woody plants [61]). Additionally, studies have demonstrated that species may not vanish from the ecosystem, but large population declines are sufficient to diminish the amount and quality of seed dispersal to such an extent that plant recruitment is no longer feasible, triggering negative feedback impacts on plant populations, community dynamics and hence ecosystem functions [62–64]. Proportionally, our analysis shows that insectivores constitute the second most sensitive guild, despite including more species loss than any other group in absolute terms (between 4–74% species loss, in Terra do Meio Mosaic, RCP 2.6, 2050, and ESEC Rio Ronuro, RCP 8.5, 2050, respectively). Regardless of the low number of studies that have been conducted, the importance of insectivorous birds in reducing plant damage from herbivory has been demonstrated in Neotropical forest canopies [65]. Although less sensitive than other guilds, nectarivores could potentially lose up to 60% of their current projected diversity (e.g., IL Kayabi and Rikbaktsá, RCP 8.5, 2050). A recent estimation found that >90% of angiosperm species in tropical communities are animal pollinated [66] and over 111 avian species pollinate plants in Brazil (effectively or potentially), but their importance in pollination is not well documented [67]. The possible impact of climate change on reducing bird-pollination services is unknown and could include decrease in gene flow among plants, as well in fruit and seed set [68]. Using knowledge of biological interactions coupled with current and expected future climate conditions is a key strategy to improve the efficiency of conservation plans in terms of restoration and strengthening landscape connectivity [69,70]. Notwithstanding, as noted previously, such information need to be more deep- and locally accessed to better evaluate the consequences for other communities of species that interact with avian species. Moreover, the species losses mentioned above did not consider species turnover (i.e., the amount of arriving species), and when it was considered, the SR in each PA did not change substantially. At first glance, this could be considered resilience if it is interpreted as a community response to perturbation, but a large part of this turnover was due to generalists. Several questions remain unanswered about "functional homogenization" in terms of if generalists represent a new degraded state, a transition that will return to the initial equilibrium, or a transition to a novel ecosystem; regardless, generalists invariably affect functioning and productivity, reducing resistance and resilience (see [71] for a review).

Our study identified a large, almost connected, and, in great part, intact block in the focal area as climatically stable for at least 20% of each FT trophic guild. Approximately the same area was forecasted for bats using the same parameters [26]. Our analysis shows that about 55% of our modelled climatically stable area overlap with currently established PAs. Despite of indigenous lands are legally not regarded as conservation units by the Brazilian law, these areas strongly ensure biodiversity protection [72,73]. Notably, the westernmost areas are the best preserved and protected, even under heavy pressure from illegal logging and mining, as well as recent government attempts to reduce protected areas (provisional measures MP 756/2016 and MP 758/2016 [74]) and undermine environmental laws to facilitate the implementation of large infrastructure projects (constitutional amendment–PEC65 [75]). On the other hand, approximately 15% of the area overlaps with degraded areas, and the remaining 30% still have natural vegetation but no protection. Together, these areas could form a large continuous block of suitable habitat for avian species. Several studies have demonstrated that large, connected, and intact ecosystems ensure the representativeness of multiple landscape gradients, ecological effectiveness of species population size, and intraspecific genetic diversity ([76] and references therein). Also, the contribution undertaken by Brazil at the 2015 Paris Conference of the Parties is to restore 120,000 km^2^ of forests by 2030. Most of this restoration should be in sites potentially suitable for as many species as possible, such as those pointed out by our models.

Several studies have already analysed and described how methodological uncertainty could complicate predicting the impacts of climate change [77–80]. In fact, each stage of the modelling method provides variation that generate uncertainties (e.g., collinearity and variable selection [81]; GCMs [82]; future emission scenarios; algorithms [83]; threshold selection [84], without mentioning the uncertainties from the data which is hard to fully explore). Recent works that have partitioned and measured the contribution of the sources of variation inherent to the models, show that the algorithms are responsible for most of the uncertainty, being greater than the climatic models and the future emission scenarios [49,77,81]. Our results are in line with those showing that our algorithms contributed the most to uncertainty when compared with emission scenarios. Although our conclusions are based upon simulation derived from only one GCM, significant efforts have been made between the IPCC's Assessment Reports to reduce biases in climate models [34] and, as above mentioned, our choice were based in recent analysis showing that CCSM4 best represent the main features over northern South America [42,43]. Here, our main goal is not to compare computational methods but instead to provide decision makers with an array of possible outcomes related to the potential impacts of climate change on avian assemblages, considering our large set of species data, the resolution of multi-algorithm, multi-timescale and multi-emission scenario and computational feasibility. There are two more important points that relate to our analysis. Firstly, the dependence of model accuracy methods on sample/species prevalence, highlighting that without reliable presence—absence data, no metrics yield proper estimates (even for TSS as recently demonstrated) [85–87]. True absences already are very tough to obtain because they demand high sampling effort to ensure their exactitude, which is much harder in the neotropics where species records’ data (true presences) have spatial, temporal and taxonomic biases [14,15]. In addition, expecting for a flawless evaluation metric is unworthy when we can use the available ones in a complementary way. Therefore, we believe that our consensus maps are robust in the projections of possible distribution/composition changes of avian assemblages in the study area. Another important point is the idiosyncratic response of each species to climate change, stressing out that different species may not react in the same way and for this reason, we focus on the general inferred patterns for guild groups. Finally, conservation actions based on our results must assume a flexible strategy (i.e., accept the risks, reassess conditions in front of new evidence, adapt/change strategy) keeping in mind managing in the face of uncertainty [69].

## Conclusions

Throughout our habitat suitability models, we show that (1) birds in SE Amazonia will be affected by climate change even under the most optimistic scenario; (2) frugivores are the most sensitive group facing climate change in coming decades which could bring consequences on seed dispersion and plant recruitment; and (3) the current set of protected areas has the potential to protect half of current projected biodiversity, and that 55% of climatically stable areas identified in this study overlapped with PAs. In the context of decision-making, our models are important in suggesting insightful conservation strategies that involve not only improving currently established PAs but also demonstrating which areas to maintain and which to restore to optimize the potential for natural processes, such as dispersal and adaptation.

## Supporting information

S1 Fig Geographic patterns of model uncertainty. Uncertainty proportion associated to algorithms and future scenarios, based on the total sum of squares.
(TIF)

S1 Table Summary of data and model performance statistics. Nrec: number of occurrence; Habitat: Forest (FT) and Open-area vegetation (OAV); Guild: Frugivores (FR), Insectivores (IN), Nectarivores (NE), Others (OT); Status: Brazilian Endemic (ED), Near Threathened (NT), Vulnerable (VU), Endangered (EN), Critically Endangered (CR), *IUCN, ‡MMA, †SEMAS-PA; Model performance: ROC, TSS, Sensitivity, Specificity; Cutoff: threshold values maximized TSS for binarization; Variables importance: mean (sd) between runs; bold numbers represent mean values higher than 30% of importance.
(XLSX)

S2 Table Protected areas. Conservation Units (CU) and Indigenous Lands (IL) and the mosaics of which they are part.
(XLSX)

S3 Table Area change. Projected species occurrence area and differences between scenarios.
(XLSX)

S4 Table Biodiversity change dynamics in protected areas. Projected species richness and species turnover in Protected Areas.
(XLSX)

S1 File The R code. occurrence records acquisition, modeling procedures and analysis.
(R) Click here for additional data file.
