# Supplementary figures and images for: Climate change impact on ecosystem functions provided by birds in southeastern Amazonia
Source: PLoS One. 2019 Apr 11;14(4):e0215229. doi: 10.1371/journal.pone.0215229 (PMC6459508; doi:10.1371/journal.pone.0215229)

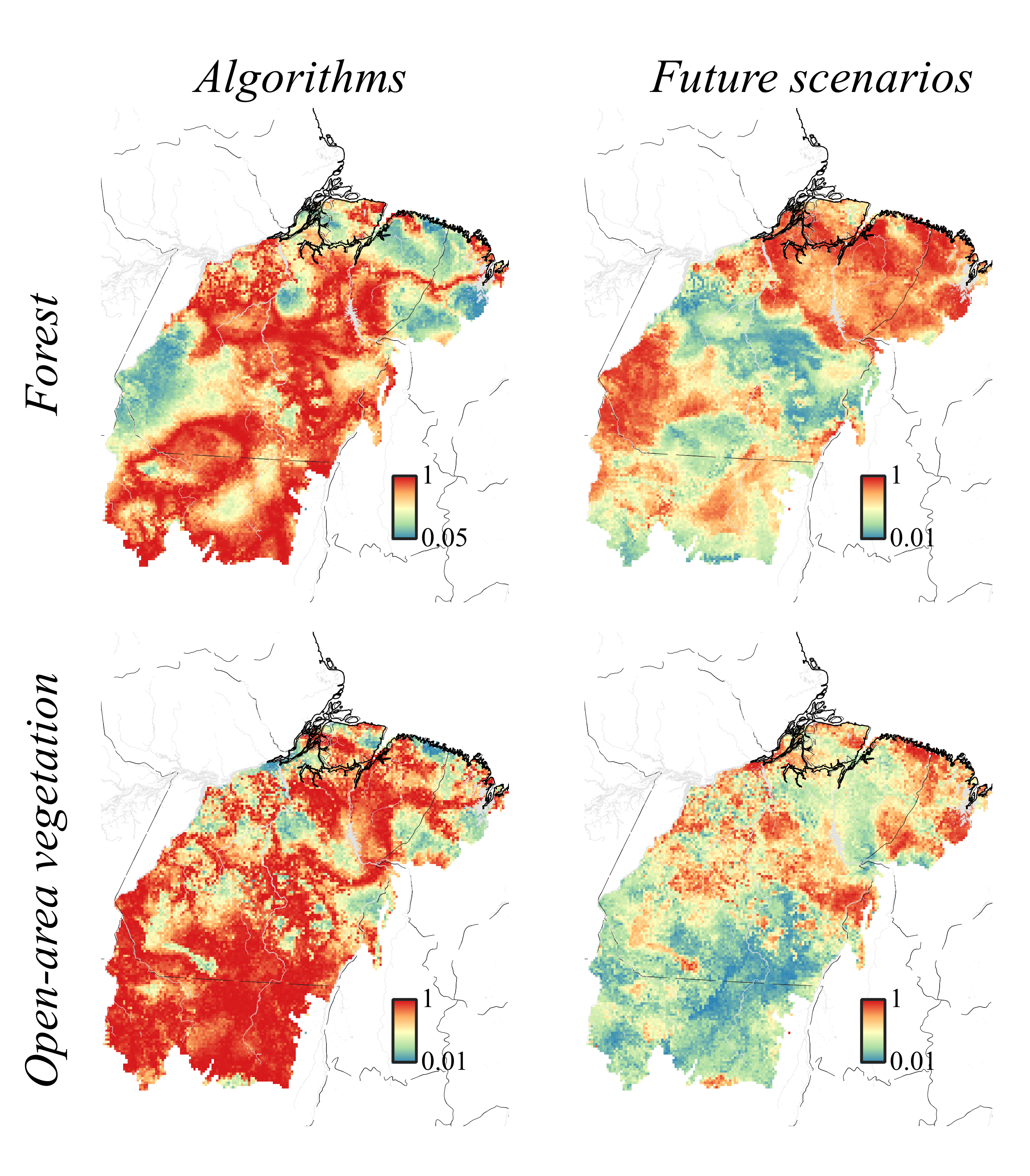

Supplement: S1 Fig — Uncertainty proportion associated to algorithms and future scenarios, based on the total sum of squares. (TIF) [file pone.0215229.s001.tif]
